# Acute and long-term outcomes in a *Drosophila melanogaster* model of classic galactosemia occur independently of galactose-1-phosphate accumulation

**DOI:** 10.1242/dmm.022988

**Published:** 2016-11-01

**Authors:** Jennifer M. I. Daenzer, Patricia P. Jumbo-Lucioni, Marquise L. Hopson, Kerry R. Garza, Emily L. Ryan, Judith L. Fridovich-Keil

**Affiliations:** Department of Human Genetics, Emory University School of Medicine, Atlanta, GA 30322, USA

**Keywords:** Galactosemia, *Drosophila*, Gal-1P, Galactose

## Abstract

Classic galactosemia (CG) is a potentially lethal inborn error of metabolism that results from the profound loss of galactose-1-phosphate uridylyltransferase (GALT), the second enzyme in the Leloir pathway of galactose metabolism. Neonatal detection and dietary restriction of galactose minimizes or resolves the acute sequelae of CG, but fails to prevent the long-term complications experienced by a majority of patients. One of the substrates of GALT, galactose-1-phosphate (Gal-1P), accumulates to high levels in affected infants, especially following milk exposure, and has been proposed as the key mediator of acute and long-term pathophysiology in CG. However, studies of treated patients demonstrate no association between red blood cell Gal-1P level and long-term outcome severity. Here, we used genetic, epigenetic and environmental manipulations of a *Drosophila melanogaster* model of CG to test the role of Gal-1P as a candidate mediator of outcome in GALT deficiency. Specifically, we both deleted and knocked down the gene encoding galactokinase (*GALK*) in control and *GALT*-null *Drosophila*, and assessed the acute and long-term outcomes of the resulting animals in the presence and absence of dietary galactose. GALK is the first enzyme in the Leloir pathway of galactose metabolism and is responsible for generating Gal-1P in humans and *Drosophila*. Our data confirmed that, as expected, loss of GALK lowered or eliminated Gal-1P accumulation in *GALT*-null animals. However, we saw no concomitant rescue of larval survival or adult climbing or fecundity phenotypes. Instead, we saw that loss of GALK itself was not benign and in some cases phenocopied or exacerbated the outcome seen in *GALT*-null animals. These findings strongly contradict the long-standing hypothesis that Gal-1P alone underlies pathophysiology of acute and long-term outcomes in *GALT*-null *Drosophila* and suggests that other metabolite(s) of galactose, and/or other pathogenic factors, might be involved.

## Abstract (precis)

**Summary:** In a GALT-deficient *Drosophila* model of classic galactosemia, Gal-1P accumulation is not required for compromised larval survival following galactose exposure or adult movement and fecundity phenotypes.

## INTRODUCTION

**Fig. 1.**
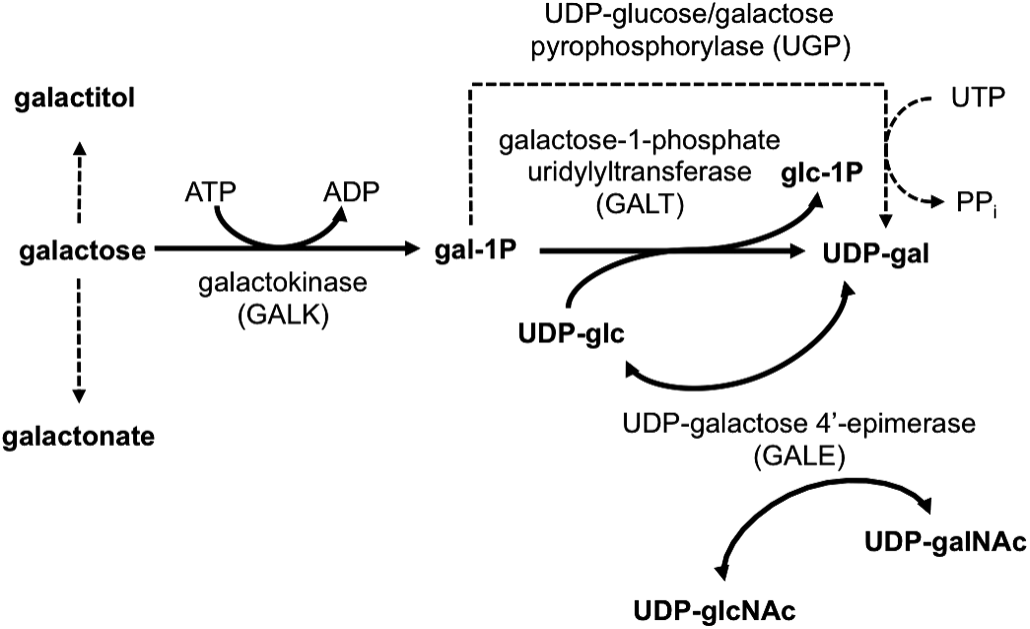
**The Leloir pathway of galactose metabolism.** In organisms ranging from bacteria to humans, galactose is metabolized by the sequential action of three enzymes: galactokinase (GALK), galactose-1-phosphate uridylyltransferase (GALT) and UDP-galactose 4′-epimerase (GALE). The dashed line indicates the UDP-glucose/galactose pyrophosphorylase-dependent bypass pathway around missing GALT.

Galactosemia is one of the most common metabolic diseases identified by newborn screening (NBS) in the United States (CDC, 2012). Classic galactosemia (CG) results from profound loss of galactose-1-P uridylyltransferase (GALT, EC 2.7.7.12) (Isselbacher et al., 1956), the second enzyme in the highly conserved Leloir Pathway of galactose metabolism (Fig. 1). Affected infants can appear normal at birth, but following exposure to high levels of galactose from lactose in breast milk or milk-based formula experience a rapid and devastating decline that can progress in days from vomiting, diarrhea and jaundice to hepatomegaly, failure to thrive, *E. coli* sepsis and neonatal death (Berry, 2014).

The early detection and rapid restriction of dietary galactose enabled by NBS for galactosemia prevents or resolves the acute and potentially lethal symptoms of CG. However, by school age most patients experience one or more of a constellation of long-term complications that include: speech, cognitive and behavioral difficulties in at least half of all patients; tremor and/or other movement problems in close to 40% of patients; growth delay in many children; low bone mineral density in many children and adults; and primary or premature ovarian insufficiency in >80% of girls and young women (Bosch, 2006; Schweitzer et al., 1993; Spencer et al., 2013; Waggoner et al., 1990; Waisbren et al., 2012). Life-long dietary restriction of galactose remains the only accepted treatment for patients with CG (Berry, 2014). However, a literature trail extending back more than 30 years documents that this treatment fails to prevent the long-term complications experienced by most patients (Gitzelmann and Steinmann, 1984; Hughes et al., 2009; Jumbo-Lucioni et al., 2012; Schweitzer et al., 1993; Segal, 1995). The mechanisms underlying acute and long-term outcomes in CG remain unclear, limiting prognosis and hampering efforts at improved intervention.

A number of intriguing hypotheses have been proposed to explain the acute and long-term pathophysiology of classic galactosemia. Many have focused on Gal-1P (e.g. Bosch, 2006; Gitzelmann and Steinmann, 1984; Tang et al., 2012), a substrate of GALT that accumulates to high levels in the red blood cells (RBCs) and tissues of affected infants, especially following milk exposure. However, repeated studies asking whether either acute neonatal or chronic childhood RBC Gal-1P levels are associated with more severe long-term outcomes among patients have failed to demonstrate a correlation (Ficicioglu et al., 2008; Hughes et al., 2009; Leslie, 2003; Schweitzer et al., 1993; Waggoner et al., 1990; Walter et al., 1999).

Studies from yeast, mice, and flies have also directly or indirectly addressed the role of Gal-1P as a candidate mediator of outcomes in GALT deficiency, and with the exception of yeast, have failed to demonstrate a causal relationship. In yeast, loss of galactokinase (*GAL1*, GALK) relieves the galactose-dependent growth restriction otherwise seen for *GALT*-null cells cultured in non-fermentable media (e.g. Douglas and Hawthorne, 1964; Ross et al., 2004). However, a *GALT*-null mouse model created in the 1990s by Leslie and colleagues failed to demonstrate any relevant acute or long-term outcomes despite accumulation of high Gal-1P levels following exposure to galactose (Leslie et al., 1996; Ning et al., 2000). A new *GALT*-null mouse, reported in 2014 by Lai and colleagues (Tang et al., 2014), demonstrated only subtle defects despite exposure to extraordinarily high levels of dietary galactose.

Prior studies using a *GALT*-null *Drosophila melanogaster* model of GALT-deficiency created in our laboratory (Kushner et al., 2010) further challenged the idea that Gal-1P accumulation underlies outcomes in GALT deficiency. For example, we found that Gal-1P levels in *GALT*-null larvae exposed to low sub-lethal dietary galactose were in the same range as those seen in larvae exposed to high lethal doses of galactose (Ryan et al., 2012), although the resulting outcomes were clearly different. We further demonstrated that exposure of *GALT*-null larvae to oxidants and anti-oxidants that modulated acute and long-term outcomes conferred their effects independently of changes to Gal-1P (Jumbo-Lucioni et al., 2013, 2014b). However, these experiments addressed the role of Gal-1P accumulation only indirectly.

Galactose, galactitol, and galactonate also accumulate in patients with CG and have been proposed as candidate mediators of disease (reviewed in Fridovich-Keil and Walter, 2008). However, until recently these other metabolites were generally discounted because they also accumulate in patients with GALK deficiency, an extremely rare condition in many populations that was long considered benign except for galactose-dependent cataracts (reviewed in Bosch et al., 2002). In 2011, that assumption was upended, however, by a report describing the outcomes of 18 patients with GALK deficiency identified by NBS in Germany (Hennermann et al., 2011). Of the 16 patients in this cohort evaluated for cognitive function, 31% were found to be intellectually disabled. Of note, these patients experienced accumulation of galactose and other metabolites such as galactitol, but did not accumulate Gal-1P. Whether the negative cognitive outcomes in these patients reflected only their GALK-deficiency could not be conclusively proved, but they did associate with continued dietary galactose exposure and did not associate with known consanguinity in the families.

Here, we used genetic deletion and RNA interference (RNAi)-mediated knockdown of *GALK* to prevent or minimize Gal-1P synthesis in *GALT*-null and control *Drosophila* and tested both larval galactose sensitivity and adult climbing and female fecundity phenotypes in the resulting animals. Our findings clearly demonstrated that loss or knockdown of *GALK* prevented or lowered accumulation of Gal-1P in *GALT*-null animals, but also that both the larval galactose sensitivity and adult phenotypes continued to occur. As expected, galactose exposure in control animals did not phenocopy these outcomes. Our findings strongly contradict the hypothesis that Gal-1P accumulation is either necessary or sufficient to cause acute larval galactose sensitivity or negative long-term outcomes in *GALT*-null *Drosophila*.

## RESULTS

### GALK loss prevents Gal-1P accumulation in *GALT*-null *Drosophila*

To test the role of Gal-1P as a candidate mediator of outcomes in GALT deficiency, we created a genetic deletion of *GALK* in *Drosophila* and crossed this allele into both *GALT+* and *GALT*-null backgrounds. As described in Materials and Methods, we created the *GALK* deletion, *dGALK^exc9^*, by imprecise excision of a P-element in a neighboring gene, *CG5068* (EY03791). This deletion removed almost the entire *GALK* coding sequence and also part of the neighboring gene, *CG5068* (Fig. S1). Maintained under normal conditions (25°C) on molasses food, flies homozygous for *dGALK^exc9^* remained both viable and fertile, although fecundity, as judged by the production of viable offspring, was reduced.

As expected, flies homozygous for *dGALK^exc9^* exhibited no detectable GALK enzyme activity but normal levels of both GALT and UDP-galactose 4′-epimerase (GALE; Table 1). Also as expected, *GALT*-null animals lacking GALK demonstrated essentially normalized levels of Gal-1P despite dietary exposure to galactose (Table 2), confirming that, as in humans, GALK in flies is the enzyme principally responsible for the generation of Gal-1P.

**Table 1.**
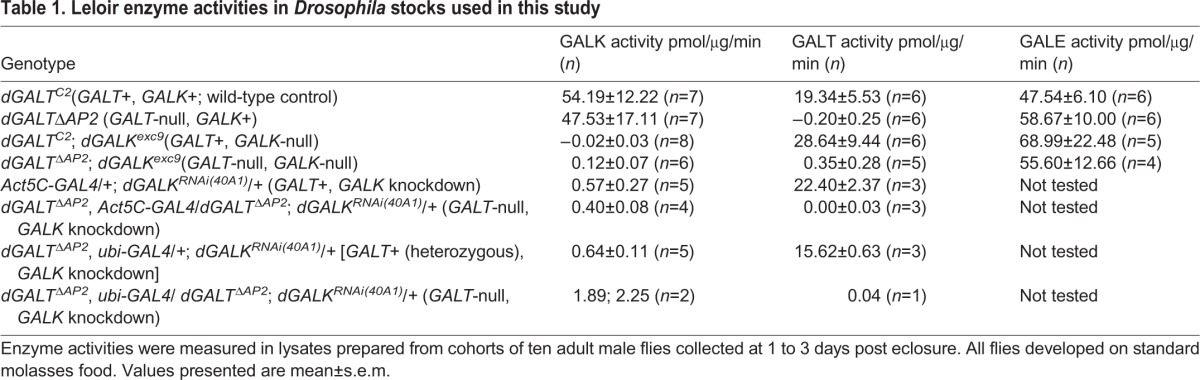
**Leloir enzyme activities in *Drosophila* stocks used in this study**

**Table 2.**
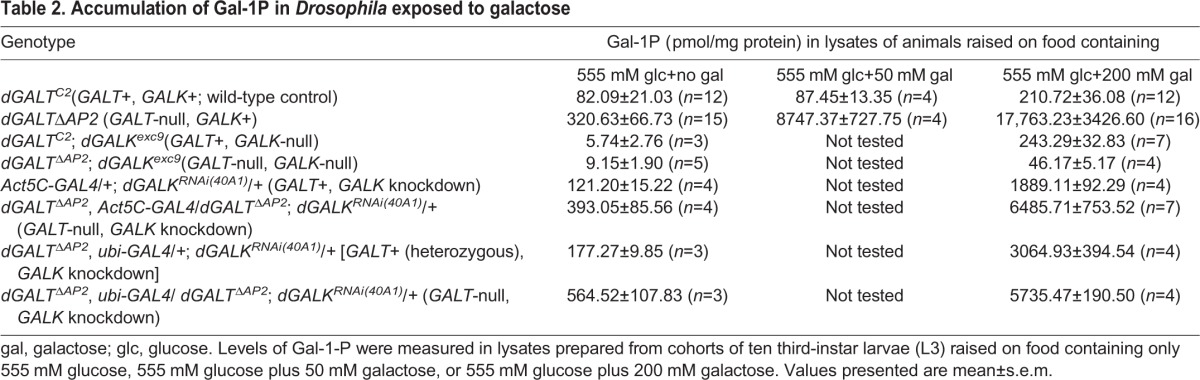
**Accumulation of Gal-1P in *Drosophila* exposed to galactose**

In separate animals, we drove a *UAS-GALK^RNAi^* allele by a broadly expressed *GAL4* driver to effectively knockdown *GALK* ubiquitously. Biochemical assays of lysates from these flies demonstrated that GALK activity was diminished to less than 4% of wild-type levels (Table 1). Metabolite studies further demonstrated that Gal-1P levels were diminished to less than 35% of the levels seen in *GALT*-null, *GALK*+ animals following galactose exposure (Table 2).

### Loss of GALK fails to rescue larval sensitivity to galactose, adult climbing and fecundity defects in *GALT*-null *Drosophila*

**Fig. 2.**
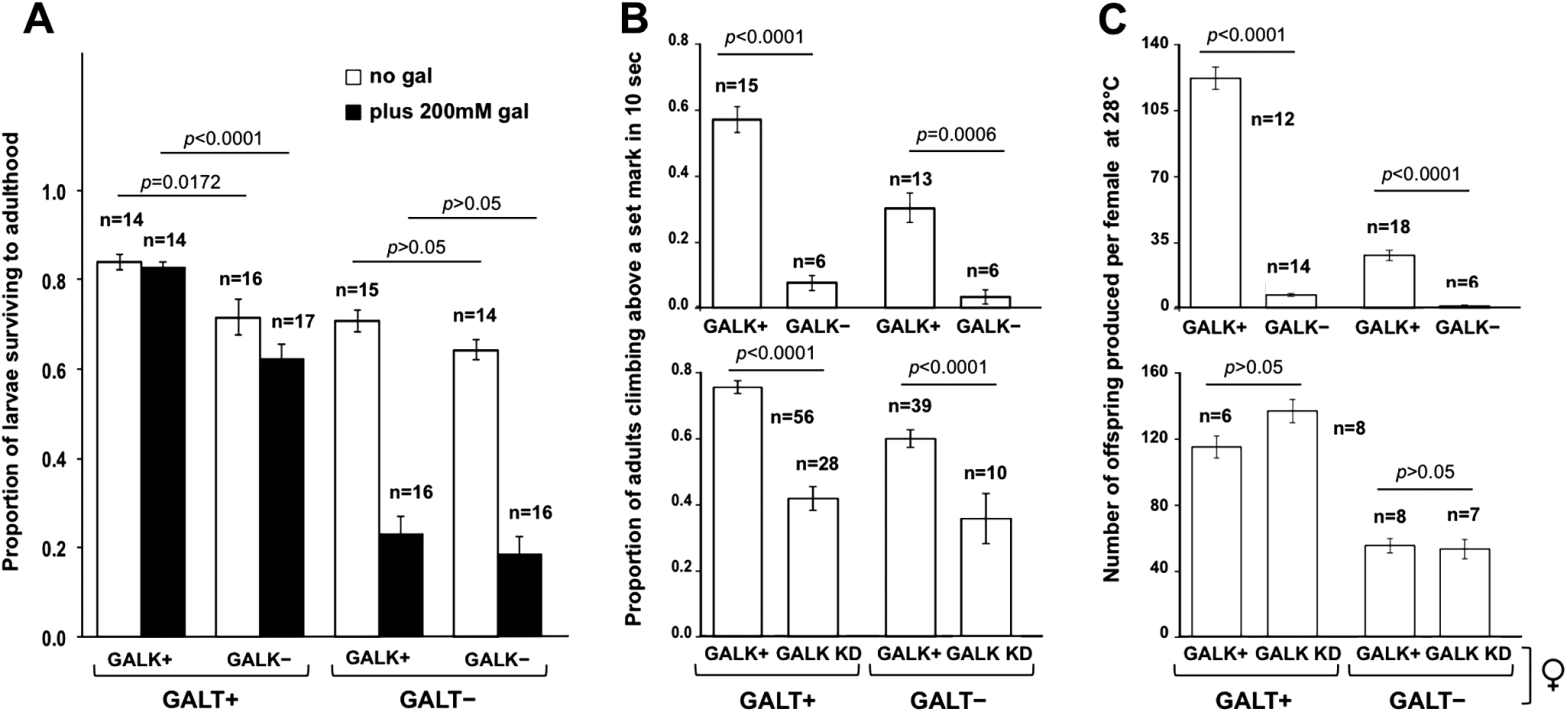
**Impact of GALK loss on acute and long-term outcomes in *GALT-*null *Drosophila*.** (A) Survival of *GALT*-null and control (*GALT+*) larvae, with and without GALK, to adulthood when raised on food containing 555 mM glucose with or without 200 mM galactose (gal). Loss of GALK diminished survival rates of both control and *GALT*-null animals in both the presence and absence of galactose. For *GALT+* animals these differences were significant: *P*=0.0172 in the absence of galactose and *P*<0.0001 in the presence of 200 mM galactose, but for *GALT*-null animals these differences were not significant (*P*>0.05 in both comparisons). (B) Climbing ability of male flies reared at 28°C in the absence of dietary galactose. Deletion of *GALK* (upper graph) not only failed to rescue this phenotype but had a significant negative impact on climbing ability for both *GALT+* (*P*<0.0001) and *GALT*-null (*P*=0.0006) flies. Knockdown (KD) of *GALK* (lower graph) also failed to rescue the climbing phenotype of *GALT*-null flies and further demonstrated a negative impact on this outcome that was independent of GALT. (C) Fecundity of female flies reared at 28°C in the absence of dietary galactose. Genetic deletion of *GALK* (upper graph) caused a striking decline in numbers of viable progeny from *GALT+* crosses (*P*<0.0001) and also exacerbated the diminished number of viable offspring resulting from crosses of *GALT*-null files (*P*<0.0001). Knockdown of *GALK* (lower graph) also failed to rescue the fecundity defect of *GALT*-null females but did not otherwise appear to impact this outcome. Values plotted represent mean±s.e.m.; the number of cohorts tested for each genotype (*n*) is indicated on the figure. *P*-values were calculated using an effects model, as described in Materials and Methods, and a *t*-test for pair-wise comparisons.

To test the role of Gal-1P accumulation on larval and adult phenotypes in *GALT*-null *Drosophila*, we compared outcomes among *GALT*-null (*dGALT^ΔAP2^*) and control (*dGALT^C2^*) larvae and adults that did encode versus those that did not encode functional GALK. The phenotypes assessed included: (1) survival of larvae to adulthood in the presence versus absence of 200 mM galactose, (2) climbing ability of 2-day-old male flies raised in the absence of galactose, and (3) the number of viable adult offspring produced at 28°C by pairings of ten young adult flies (five females of defined genotype and five wild-type males) all raised in the absence of dietary galactose. As presented (Fig. 2), loss of GALK failed to rescue the negative outcomes of *GALT*-null animals assessed for each of these phenotypes, and in some cases GALK loss itself appeared deleterious to outcome.

Specifically, whereas loss of GALT clearly compromised survival rates of larvae in the presence of 200 mM galactose, loss of GALK alone only had a small impact. Loss of GALK slightly lowered survival rates of *GALT+* animals in both the absence (*P*=0.0172) and presence (*P*<0.0001) of 200 mM galactose (Fig. 2A). Small decreases in survival of *GALT*-null, *GALK*-null animals were also evident in both the absence and presence of galactose, but these differences were not significant (*P*>0.05 in both comparisons; Fig. 2A).

Loss of GALK had a larger negative impact on the ability of young adult male flies, raised at 28°C in the absence of dietary galactose, to climb above a set mark in 10 s (Fig. 2B). This was true for both *GALT+* (*P*<0.0001) and *GALT*-null (*P*=0.0006) flies (Fig. 2B, upper graph). Finally, the compromised ability of *GALT*-null females to produce viable offspring when paired with wild-type males and reared at 28°C in the absence of dietary galactose was diminished by more than 50-fold rather than mitigated by loss of GALK (Fig. 2C, upper graph). Notably, deletion of *GALK* alone also caused a striking decline of more than 14-fold in the number of viable progeny produced by *GALT*+ females paired with wild-type males at 28°C (*P*<0.0001, Fig. 2C, upper graph). In all cases, animals lacking GALT accumulated Gal-1P unless GALK was also missing, in which case Gal-1P did not accumulate to substantial levels (Table 2).

As a further test of the roles of GALK and Gal-1P in modifying adult climbing and female fecundity outcomes, we combined a *GALK^RNAi^* allele called *dGALK^RNAi(40A1)^*, created previously in our laboratory, with a broadly expressed *GAL4* driver to knockdown expression of *GALK* in *GALT*+ and *GALT*-null *Drosophila.* Biochemical assays performed in adult animals demonstrated that the knockdown was effective; animals expressing the *GALK^RNAi^* allele with either of the two *GAL4* drivers used in this study (*Act5C-GAL4* and *ubi-GAL4*) demonstrated less than 4% of the wild-type levels of GALK (Table 1). Of note, whereas this substantial loss of GALK did lessen the accumulation of Gal-1P in *GALT*-null animals exposed to 200 mM galactose, the decrease was not proportional. Specifically, despite a greater than 25-fold decrease in GALK activity, Gal-1P accumulation decreased by only around three-fold (from 17,763.23±3426.60 to 6485.71±753.52, *Act5C* driver, or 5735.47±190.50, *ubi* driver; Table 2). We also noted an increase in Gal-1P accumulation in animals that were either *GALT*+ (1889.11±92.29) or heterozygous for *GALT-*deletion (3064.93±394.54) upon knockdown of *GALK* and exposure to 200 mM galactose; by comparison, the level of Gal-1P seen in *GALT*+ *GALK*+ animals exposed to 200 mM galactose was only 210.72±36.08 (Table 2). The meaning of this apparent Gal-1P increase despite a >25-fold knockdown of *GALK* remains unclear.

As illustrated in Fig. 2B, lower graph, knockdown of *GALK* compromised the climbing ability of both *GALT*+ and *GALT*-null flies, though by only about a factor of two rather than the factors of eight and ten, respectively, seen with *GALK* deletion. Control (*GALK+*) animals in these experiments carried either the *UAS-GALK^RNAi^* allele in the absence of driver, or the *Act5C*-*GAL4* driver alone, confirming that both were required together to see the effect.

As illustrated in Fig. 2C, bottom graph, unlike genetic deletion, knockdown of *GALK* had no significant effect on the female fecundity of either *GALT+* (*P*>0.05) or *GALT*-null (*P*>0.05) flies. Again, control (*GALK*+) animals in these experiments carried either the *UAS-GALK^RNAi^* allele in the absence of driver, or driver (*Ubi*-*GAL4*) alone.

### Exposure of *GALT*-null *Drosophila* to a low level of galactose causes accumulation of Gal-1P, but has little impact on larval galactose sensitivity or adult phenotypes

**Fig. 3.**
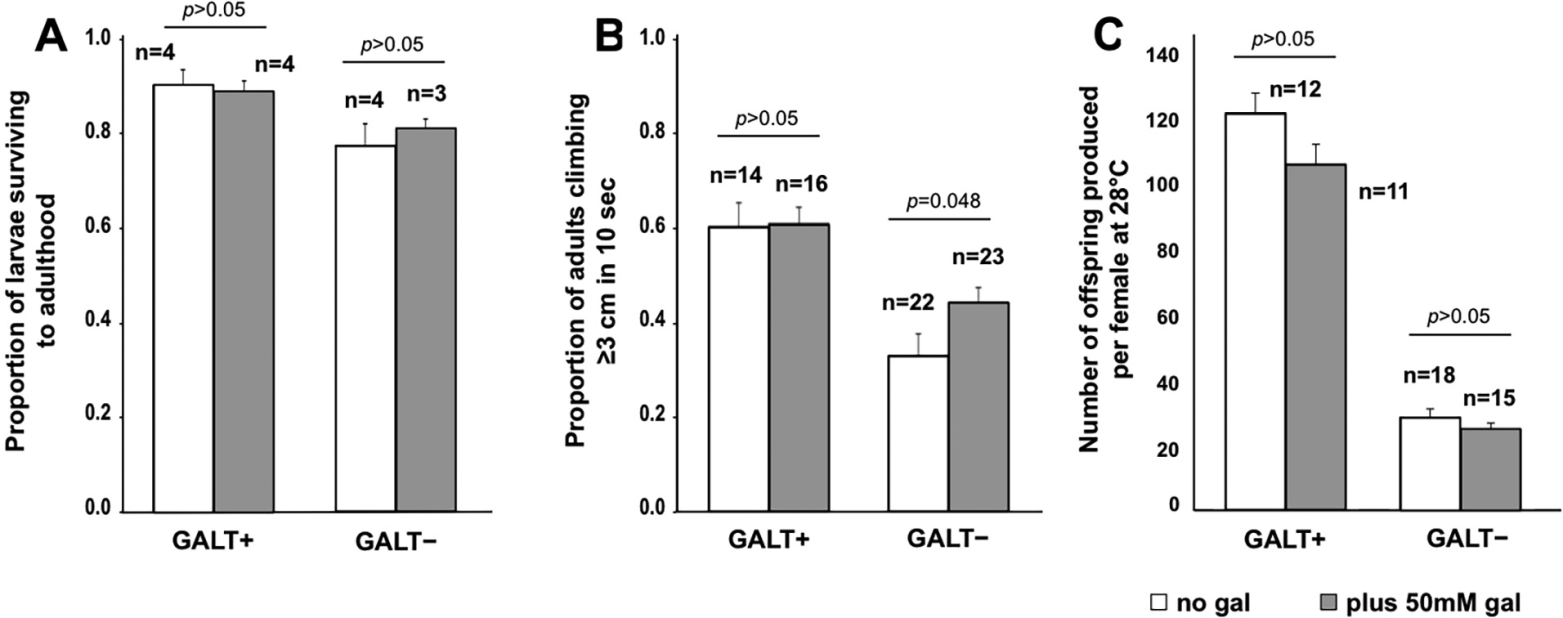
**Impact of low-level galactose exposure on acute and long-term outcomes in *GALT*-null *Drosophila*.** (A) Survival of *GALT*-null and control (*GALT+*) larvae to adulthood when raised on food containing 555 mM glucose with or without 50 mM galactose (gal). These differences were not significant (*P*>0.05; both comparisons). (B) Climbing ability of adult flies reared at 28°C in the presence versus absence of 50 mM dietary galactose. This low level of dietary galactose exposure had no apparent impact on climbing ability of *GALT+* flies and slightly improved climbing ability of the *GALT*-null flies (*P*=0.048). (C) Fecundity of female flies reared and tested at 28°C in the presence versus absence of 50 mM dietary galactose. We saw no significant impact of low-level galactose exposure on female fecundity of control animals (*P*>0.05) or *GALT*-null animals (*P*>0.05). Values plotted represent mean±s.e.m.; the number of cohorts tested for each genotype (*n*) is indicated on the figure. *P*-values were calculated using an effects model, as described in Materials and Methods, and a *t*-test for pair-wise comparisons.

As an alternative approach to testing the potential relationship between Gal-1P accumulation and outcomes in *GALT*-null *Drosophila*, we exposed both *GALT*-null and control animals to food containing 50 mM rather than 200 mM galactose (Fig. 3). Previously, we demonstrated that 50 mM galactose is sufficient to cause elevated Gal-1P in *GALT*-null *Drosophila*, but not larval lethality or climbing defects as measured using a countercurrent device (Ryan et al., 2012). Here, we repeated those experiments using a simple climbing assay and also extended the experiment to include female fecundity. As illustrated in Table 2 and Fig. 3A-C, exposure of *GALT*-null *Drosophila* to 50 mM galactose was sufficient to cause a dramatically elevated Gal-1P (Table 2) but had little, if any, impact on larval survival or adult outcomes. If anything, the low-level dietary galactose exposure partially relieved the climbing defect evident in *GALT*-null flies relative to controls (Fig. 3B); this difference was significant (*P*=0.048). We saw no significant impact of low-level galactose exposure on the fecundity of either *GALT+* (*P*>0.05) or *GALT*-null (*P*>0.05) female flies (Fig. 3C).

### Maternal loading of GALK

Finally, to address the timing of larval sensitivity to loss of GALK, we repeated the experiments presented in Fig. 2A, but set up the crosses so that resulting embryos either did or did not receive maternal loading of GALK (see Materials and Methods). Specifically, eggs produced by mothers heterozygous for *dGALK^exc9^* would be expected to include trace maternally loaded GALK whereas eggs produced by *GALK*-null mothers would not. This experimental design therefore tested the role of GALK in early embryogenesis.

**Fig. 4.**
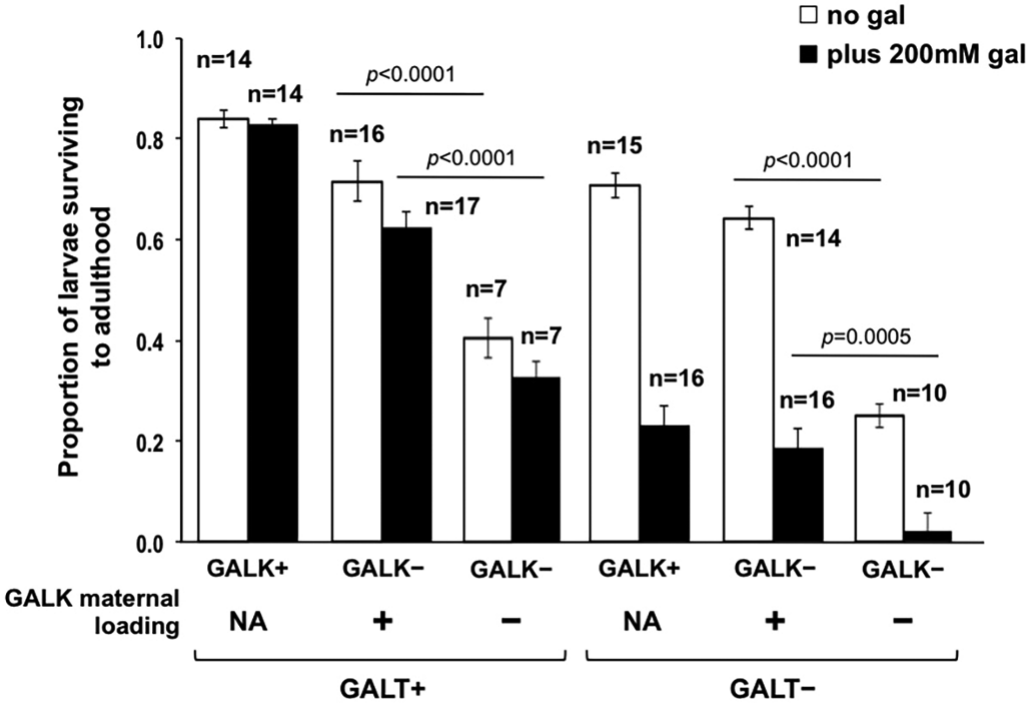
**Impact of maternal loading for GALK on survival of larvae to adulthood in the presence versus absence of 200 mM dietary galactose.** As indicated, *GALT+*, *GALK*-null larvae that received maternally loaded GALK survived to a greater extent in both the absence and presence of dietary galactose (gal) than did their counterparts that did not receive GALK maternal loading (*P*<0.0001 for both comparisons). In contrast, loss of maternally loaded GALK compromised survival of *GALT*-null *GALK*-null larvae by close to three-fold in the absence of galactose (*P*<0.0001) and by close to 10-fold in the presence of galactose (*P*=0.0005). NA, not applicable. Values plotted represent mean±s.e.m.; the number of cohorts tested for each genotype (*n*) is indicated on the figure. *P*-values were calculated using an effects model, as described in Materials and Methods, and a *t*-test for pair-wise comparisons.

As illustrated in Fig. 4, loss of maternally loaded GALK compromised survival of *GALT+ GALK-*null larvae by about two-fold in both the absence and presence of dietary galactose (*P*<0.0001 for both comparisons). The effect was accentuated in genetically *GALT*-null *GALK*-null larvae, so that loss of maternally loaded GALK compromised survival of *GALT*-null *GALK*-null larvae by close to three-fold in the absence of galactose (*P*<0.0001) and by close to ten-fold in the presence of galactose (*P*=0.0005). This result demonstrated an important role for GALK at the earliest stages of *Drosophila* development in the presence of GALT, and an apparent synergism with loss of GALT.

## DISCUSSION

Newborn screening for galactosemia coupled with rapid dietary restriction of galactose has saved the lives of thousands of infants born with CG in the United States in the past 50 years (Pyhtila et al., 2015). However, the long-term outcomes of those infants with CG who do survive remain challenging and uncertain, in large part because we still do not fully understand the pathophysiology of this disease. The GALT substrate, Gal-1P, which accumulates in individuals with CG but not controls, was long presumed instrumental in the etiology of both acute and long-term patient outcomes; however, that presumption has been contradicted by decades of study involving both humans (Ficicioglu et al., 2008; Hughes et al., 2009; Leslie, 2003; Schweitzer et al., 1993; Waggoner et al., 1990; Walter et al., 1999) and animal models (Leslie et al., 1996; Ning et al., 2000).

Here, we applied our previously described *Drosophila melanogaster* model of CG (Kushner et al., 2010) in experiments testing whether Gal-1P accumulation is either necessary or sufficient for acute larval galactose sensitivity and two adult phenotypes associated with GALT deficiency. Our results clearly demonstrate that loss of GALK, which effectively prevented the accumulation of Gal-1P, failed to prevent or even mitigate the negative acute and long-term phenotypes we assessed. In some cases, loss of GALK alone appeared to phenocopy negative outcomes associated with GALT deficiency (e.g. Fig. 2B); in other cases, it exacerbated existing negative outcomes in *GALT*-null animals (e.g. Fig. 2B,C). This exacerbation was especially evident very early in development, as indicated by the apparent synergy between GALT deficiency and loss of maternally loaded GALK (Fig. 4). Further, low-level galactose exposure caused elevated Gal-1P in *GALK+* animals but failed to elicit or exacerbate the negative acute and long-term outcomes associated with GALT deficiency (Fig. 3). Taken together, our results demonstrate that Gal-1P accumulation is neither necessary nor sufficient for the acute larval galactose sensitivity or adult climbing and female fecundity outcomes of *Drosophila* tested here.

One possible explanation for the apparent synergy between loss of GALT and loss of GALK is that when GALK is present, although the Leloir pathway is blocked by loss of GALT, the UDP-glucose/galactose pyrophosphorylase (UGP)-dependent pathway of galactose metabolism (dashed line in Fig. 1) remains active, providing a limited bypass around the GALT block. When GALK is also absent, preventing the synthesis of Gal-1P, which is a UGP substrate, both the Leloir and bypass pathways are blocked. This ‘double block’ should serve to exacerbate the accumulation of galactose and its non-Leloir derivatives (e.g. galactitol and galactonate) in *GALT*-null, *GALK*-null animals. As a preliminary test of this hypothesis, we measured the accumulation of galactose in *GALT*-null animals with and without *GALK* deletion (*dGALK^exc9^*) following exposure to 200 mM galactose. As predicted, the level of galactose was higher in the *GALK*-null animals by almost a factor of two (6459.14±909.61, *n*=3, in the *GALK*-null animals versus 3850.19±675.18, *n*=3, in the *GALK*+ animals).

On the surface, our findings presented here appear to contradict a recent report by Jumbo-Lucioni and colleagues who used the *dGALT^ΔAP2^* and the *dGALK^exc9^* alleles provided by our laboratory to test the role of GALK loss on synapse morphology and a larval movement phenotype in GALT-deficient *Drosophila* (Jumbo-Lucioni et al., 2014a). Their report stated that homozygosity for the *dGALK^exc9^* allele relieved many of the phenotypes they measured. However, the larval outcomes described by Jumbo-Lucioni et al. were different from the phenotypes measured here. Of note, some of the larval phenotypes assessed by Jumbo-Lucioni and colleagues (Jumbo-Lucioni et al., 2014a) were also apparently phenocopied in control (*GALT+*) animals exposed to galactose, which is contrary to the larval and adult phenotypes we have observed previously and describe here (Kushner et al., 2010; Ryan et al., 2012). To be clear, even exposure of wild-type *Drosophila* to high (200 mM) levels of galactose does not mimic the larval death and adult climbing and female fecundity outcomes described here (Fig. 2A, and data not shown) and previously by our group (e.g. Kushner et al., 2010; Ryan et al., 2012). Given that normal human breast milk contains approximately 170 mM galactose (in the form of lactose) (Lubetzky et al., 2015), phenocopying of patient outcomes in controls exposed to galactose is also clearly at odds with the experience in humans.

One potential caveat to the experiments described here utilizing the *dGALK^exc9^* allele is that in addition to deleting *dGALK* this excision also removes part of a neighboring gene, *CG5068*. Little is known about the function of *CG5068*, but from homology studies it is predicted to encode a carboxylic ester hydrolase ostensibly involved in protein demethylation (http://flybase.org/reports/FBgn0035951.html). Experiments reported by Mummery-Widmer and colleagues state that knockdown of *CG5068* results in animals that are viable and fertile with no recognized phenotypes (Mummery-Widmer et al., 2009). Furthermore, we observed that RNAi-mediated knockdown of *GALK* in animals genetically wild-type for *GALK* and *CG5068* also failed to relieve the negative climbing and female fecundity phenotypes associated with GALT-deficiency. Knockdown of *GALK* alone also resulted in a partial climbing defect but not a female fecundity defect. Although it is possible that the quantitative differences observed between climbing and female fecundity outcomes of *GALK* deletion and knockdown animals reflect the presence of residual GALK activity in the knockdown animals, it also remains possible that some of the negative outcomes associated with homozygosity for the *dGALK^exc9^* allele might reflect loss of *CG5068* as well as loss of *GALK*.

### If not Gal-1P, then what?

Prior studies (e.g. Douglas and Hawthorne, 1964; Ross et al., 2004) clearly document that loss of *GALK* relieves the galactose-dependent growth phenotype of *GALT*-null yeast; however, the data presented here clearly demonstrate that result is not transferrable to at least three whole-organism phenotypes of GALT deficiency in *Drosophila*. The reason for this difference remains unclear, but the potential implication for other metazoans, including humans, is unavoidable.

If the accumulation of Gal-1P is neither necessary nor sufficient to account for the negative outcomes associated with GALT deficiency in *Drosophila*, then what is? Studies from patients and model systems document that loss of GALT results not only in the accumulation of Gal-1P but also in elevated levels of galactose, galactitol and galactonate (Fig. 1, reviewed in Fridovich-Keil and Walter, 2008). Studies from a mouse model of GALK deficiency (Ai et al., 2000) documented that galactitol, produced from galactose by aldose reductase expressed in the lens, was responsible for the cataract formation observed in those animals. That both deletion and knockdown of *GALK* failed to mitigate most of the phenotypes studied here, and in fact phenocopied or exacerbated some of them, suggests that galactose, galactitol or other galactose metabolites – or a combination of metabolites and other factors such as oxidative stress (Jumbo-Lucioni et al., 2013) or perturbed glycosylation resulting from altered levels or ratios of UDP-sugars (reviewed in Fridovich-Keil and Walter, 2008) – might underlie the pathophysiology of acute and long-term outcomes in GALT deficiency.

## MATERIALS AND METHODS

### Fly stocks and maintenance

Unless otherwise noted, stocks were maintained at 25°C with 60–70% humidity on a molasses-based food containing 43.5 g/l cornmeal, 17.5 g/l yeast extract, 8.75 g/l agar, 54.7 ml/l molasses, 10 ml propionic acid and 14.4 ml/l tegosept mold inhibitor (10% w/v in ethanol). For most experiments in which the levels and types of sugar were to be varied we used a glucose-based food [5.5 g/l agar, 40 g/l yeast, 90 g/l cornmeal, 100 g/l glucose, 10 ml/l propionic acid and 14.4 ml/l tegosept mold inhibitor (10% w/v in ethanol)] (Honjo and Furukubo-Tokunaga, 2005) supplemented with galactose, as indicated. For female fecundity experiments, we either did or did not add galactose to molasses food. All fly stocks were obtained from the Bloomington *Drosophila* Stock Center at Indiana University unless otherwise noted.

### Creation of a *dGALK^exc9^* deletion allele by imprecise P-element excision

We created the *dGALK^exc9^* deletion allele by imprecise excision of an existing P-element insertion, *EY03791*, located less than 1 kb upstream of the *dGALK* start site and within a neighboring gene, *CG5068*. The P-element was mobilized by transient expression of the *Δ2-3* transposase enzyme in the male germ line, according to standard methods (Ryder and Russell, 2003). Flies carrying excision alleles were identified by loss of the associated mini-*w+* marker (white eyes) and were screened biochemically by performing a GALK enzyme assay in lysates. One imprecise excision, designated as *dGALK^exc9^*, demonstrated complete loss of detectable GALK activity. The breakpoints of the 2990 bp deletion were determined by PCR amplification with primers 5′-TAGTGCCTCCATGGCTGTGC-3′ and 5′-GTCCACAGCAATGCGCATGC-3′ followed by sequencing of the junction fragment.

### Generation of animals experiencing *GALK* knockdown

*UAS-GALK^RNAi^* lines were created in our laboratory by cloning a *GALK* RNAi fragment made by PCR amplification of wild-type *Drosophila* genomic DNA (using primers 5′-CCGCGAATTCAGAATCGAGCTTCCAAAGAGTGG-3′ and 5′-CCGCGAATTCAGCACGTTGACGCAGCTTGAAC-3′) into the expression vector *pSYMP* using *EcoR1* sites*.* Transgenic lines were created by injecting this plasmid together with *P/TS129A.Act5C* (Beall et al., 2002). Injections were performed at BestGene Inc. (https://www.thebestgene.com/) and resulting transformants were identified by the presence of red eye color. The genomic insertions were mapped by standard methods. The allele used here was designated *GALK^RNAi(40A1)^*.

Knockdown of *GALK* was achieved by crossing flies carrying *GALK^RNAi(40A1)^* to flies carrying an appropriate *GAL4* driver. For climbing experiments, the driver used was *P{Act5C-GAL4}25FO1*; for fecundity experiments the driver used was *P{Ubi-GAL4.U}2*. Both drivers were recombined with *GALT^ΔAP2^* to test *GALK* knockdown in *GALT-null* flies.

For fecundity experiments involving *GALK* knockdown, flies denoted ‘*GALT*+’ were actually heterozygotes with one wild-type chromosome 2 and one chromosome 2 that carried the *GALT^ΔAP2^* deletion allele. Specifically, *GALK* knockdown flies carried a recombinant chromosome 2 with both *GALT^ΔAP2^* and *Ubi-GAL4*, whereas ‘control’ flies lacking driver carried a chromosome 2 with *GALT^ΔAP2^.* In all cases, these flies had maternal loading of *GALT*. To ensure heterozygosity for the *GALT^ΔAP2^* deletion did not impact fecundity, we quantified the numbers of viable offspring produced by *GALT*+/+ versus *GALT*+/*GALT^ΔAP2^* females in parallel crosses; there was no significant difference (data not shown).

### Quantifying enzymes and metabolites

GALK, GALT and GALE enzyme activity assays were performed on adult male flies as described previously (Sanders et al., 2010). *Drosophila* larvae intended for analysis of galactose metabolites were raised on food containing either 555 mM glucose as the sole sugar or 555 mM glucose plus the indicated level of galactose (e.g. 50 mM or 200 mM). *Drosophila* adults intended for analysis of galactose metabolites were raised on standard molasses food. The newly eclosed adults were placed on food containing either 555 mM glucose as the sole sugar or 555 mM glucose plus 200 mM galactose and harvested after 48 h. Cohorts of third-instar larvae or adults were harvested and metabolites extracted as described previously (Sanders et al., 2010); metabolites were separated and quantified using a Dionex HPLC, essentially as described previously (Ross et al., 2004) with the following changes: measurement of galactose was performed using an MA1 column (Thermofisher) with buffers A (1 M sodium hydroxide) and B (15 mM sodium hydroxide). Galactose was separated using an isocratic procedure with a flow rate of 0.4 ml/min and buffer concentrations of 50% buffer A and 50% buffer B for 30 min. For all samples, 20 µl was injected into a 25 µl injection loop.

### Quantifying survival of *Drosophila* larvae to adulthood on different foods

We measured the impact of dietary galactose exposure on survival of developing *Drosophila* as described previously (Jumbo-Lucioni et al., 2013). Briefly, parents of the desired genotypes were allowed to mate and deposit eggs on grape juice and agar medium for 24 h. After an additional 24 h, cohorts of 20 first-instar larvae were collected under a dissecting microscope with a small spatula and transferred to 0.5 ml microfuge tubes pre-loaded with the desired food. Once loaded with larvae, each microfuge tube was positioned into the bottom of a 12×55 mm clear polystyrene vial. Vials were then plugged with cotton and maintained at 25°C with 60-70% humidity, and inspected daily. The number of surviving adult flies in each vial was recorded for 9 days beyond appearance of the first adult.

### Quantifying climbing ability of adult *Drosophila*

Newly eclosed male flies, which developed at 28°C, were collected and maintained in cohorts of 9–11 animals per vial on standard molasses food. After 48 h, each cohort was tapped into a tall glass test tube marked with a blue line at either 3 cm or 8 cm above the base and plugged with cotton. Because we found that, as a group, flies carrying the *mini-white* marker climbed better than *w–* flies, we required them to climb the greater distance (8 cm). Only flies matched in terms of presence or absence of *mini-white* were compared*.* Specifically, genetic mutants and controls, all of which were *w–*, were required to climb 3 cm (Fig. 2B, top panel, and Fig. 3B), whereas flies used to test the effects of *GALK* knockdown, which all carried the *mini-white* marker, were required to climb 8 cm (Fig. 2B, bottom panel). Flies were not anesthetized with CO_2_ for this procedure.

Just prior to *t*=0, tubes were dropped through a chute from a height of 35 cm onto a rubber pad, knocking all the flies to the bottom. These flies were then given 10 s to climb up the sides of the tube and the number that climbed up to or past the set mark was recorded. Each cohort was tested three times and the average was converted into a proportion that was used as the climbing score for that cohort.

### Quantifying female fecundity in *Drosophila*

We quantified female fecundity of specific genotypes under given environmental conditions by counting the numbers of viable offspring produced by cohorts of newly eclosed female flies crossed to virgin wild-type males. The male flies in all crosses were 1-3 days old, and all had developed at 25°C. The female flies used in all fecundity experiments were reared at 28°C. For the study illustrated in Fig. 2C, we tested cohorts of ten flies (five male and five female) crossed in vials with standard molasses food. For the study illustrated in Fig. 3C, we tested cohorts of ten flies (five male and five female) crossed in vials of food containing either standard molasses food or standard molasses food plus 50 mM galactose, as indicated. In all experiments, vials were incubated at 28°C and adults were tapped to a fresh vial every 1-3 days, for a total of 10 days, to prevent overcrowding of the offspring. The numbers of viable adult offspring emerging in each vial were counted and summed for 8 days from the day the first fly eclosed. Each vial was also inspected every few days for the presence of both eggs or embryos and larvae. We noted that whereas the single (*GALT-*null or *GALK*-null) and double mutant flies (*GALT*-null *GALK*-null) all produced few adult offspring in this assay, they did lay a substantial number of eggs. However, unlike their wild-type counterparts, the vast majority of these eggs or embryos failed to hatch into visible larvae, suggesting either that they were never fertilized or that they died early in embryogenesis prior to hatching. We are currently working to define the nature and mechanism of this apparent female fecundity defect.

### Preparing cohorts of *GALK*-null larvae that do versus do not have maternal loading for GALK

Larvae lacking maternal loading for *GALK* were derived from crosses of *GALK*-null males and females. Larvae that were genetically *GALK*-null but had received maternal loading for *GALK* were derived from crosses of *GALK*-null males with females carrying one *dGALK^exc9^* chromosome 3 over a *GALK+* balancer chromosome 3 that also encoded GFP. L1 larvae from both types of crosses were subjected to UV sorting and only ‘dark’ larvae lacking GFP signal were selected for use.

### Statistical analyses

Statistical analyses were performed using JMP-SAS software. Survival, climbing and fecundity data were analyzed using linear regression models that included both genotype and diet (when diet was varied) as independent variables and also included the genotype by diet interaction. Post-hoc comparisons were performed on the least-square means to determine significant differences between groups. Because we have previously established the effects of feeding *GALT+* and *GALT*-null flies a diet containing 200 mM galactose (Kushner et al., 2010), unless otherwise noted, here we only made statistical comparisons between groups of flies where the *GALT* genotype and diet were held constant, so the sole difference was presence or absence of *GALK*. The criterion for statistical significance was *P*<0.05, and *P*-values were adjusted for multiple comparisons using a simple Bonferroni correction. All data are presented as mean±s.e.m.

## References

[DMM022988C1] Ai Y., Zheng Z., O'Brien-Jenkins A., Bernard D. J., Wynshaw-Boris T., Ning C., Reynolds R., Segal S., Huang K. and Stambolian D. (2000). A mouse model of galactose-induced cataracts. *Hum. Mol. Genet.* 9, 1821-1827. 10.1093/hmg/9.12.1821 10915771

[DMM022988C2] Beall E. L., Mahoney M. B. and Rio D. C. (2002). Identification and analysis of a hyperactive mutant form of Drosophila P-element transposase. *Genetics* 162, 217-227. 12242235 10.1093/genetics/162.1.217 PMC1462248

[DMM022988C3] Berry G. (2014). Classic galactosemia and clinical variant galactosemia. In *GeneReviews^®^* (ed. Pagon R., Adam M., Ardinger H., Bird T., Dolan C., Fong C., Smith R. and Stephens K.). Seattle, WA: University of Washington.

[DMM022988C4] Bosch A. M. (2006). Classical galactosaemia revisited. *J. Inherit. Metab. Dis.* 29, 516-525. 10.1007/s10545-006-0382-0 16838075

[DMM022988C5] Bosch A. M., Bakker H. D., van Gennip A. H., van Kempen J. V., Wanders R. J. A. and Wijburg F. A. (2002). Clinical features of galactokinase deficiency: a review of the literature. *J. Inherit. Metab. Dis.* 25, 629-634. 10.1023/A:1022875629436 12705493

[DMM022988C6] CDC (2012). CDC grand rounds: newborn screening and improved outcomes. In *Morbidity and Mortality Weekly Report (MMWR)*, vol. 61, pp. 390-393. Atlanta, GA: Centers for Disease Control and Prevention. 22647744

[DMM022988C7] Douglas H. C. and Hawthorne D. C. (1964). Enzymatic expression and genetic linkage of genes controlling galactose utilization in saccharomyces. *Genetics* 49, 837-844. 14158615 10.1093/genetics/49.5.837 PMC1210618

[DMM022988C8] Ficicioglu C., Hussa C., Yager C. and Segal S. (2008). Effect of galactose free formula on galactose-1-phosphate in two infants with classical galactosemia. *Eur. J. Pediatr.* 167, 595-596. 10.1007/s00431-007-0520-1 17554561

[DMM022988C9] Fridovich-Keil J. and Walter J. (2008). Galactosemia. In *The Online Metabolic & Molecular Bases of Inherited Disease* (ed. Valle D., Beaudet A., Vogelstein B., Kinzler K., Antonarakis S. and Ballabio A.), pp. http://www.ommbid.com/: McGraw Hill.

[DMM022988C10] Gitzelmann R. and Steinmann B. (1984). Galactosemia: how does long-term treatment change the outcome? *Enzyme* 32, 37-46. 6479120 10.1159/000469448

[DMM022988C11] Hennermann J. B., Schadewaldt P., Vetter B., Shin Y. S., Mönch E. and Klein J. (2011). Features and outcome of galactokinase deficiency in children diagnosed by newborn screening. *J. Inherit. Metab. Dis.* 34, 399-407. 10.1007/s10545-010-9270-8 21290184

[DMM022988C12] Honjo K. and Furukubo-Tokunaga K. (2005). Induction of cAMP response element-binding protein-dependent medium-term memory by appetitive gustatory reinforcement in Drosophila larvae. *J. Neurosci.* 25, 7905-7913. 10.1523/JNEUROSCI.2135-05.2005 16135747 PMC6725454

[DMM022988C13] Hughes J., Ryan S., Lambert D., Geoghegan O., Clark A., Rogers Y., Hendroff U., Monavari A., Twomey E. and Treacy E. P. (2009). Outcomes of siblings with classical galactosemia. *J. Pediatr.* 154, 721-726. 10.1016/j.jpeds.2008.11.052 19181333

[DMM022988C14] Isselbacher K. J., Anderson E. P., Kurahashi K. and Kalckar H. M. (1956). Congenital galactosemia, a single enzymatic block in galactose metabolism. *Science* 123, 635-636. 10.1126/science.123.3198.635 13311516

[DMM022988C15] Jumbo-Lucioni P. P., Garber K., Kiel J., Baric I., Berry G. T., Bosch A., Burlina A., Chiesa A., Pico M. L., Estrada S. C. et al. (2012). Diversity of approaches to classic galactosemia around the world: a comparison of diagnosis, intervention, and outcomes. *J. Inherit. Metab. Dis.* 35, 1037-1049. 10.1007/s10545-012-9477-y 22450714 PMC3774053

[DMM022988C16] Jumbo-Lucioni P. P., Hopson M. L., Hang D., Liang Y., Jones D. P. and Fridovich-Keil J. L. (2013). Oxidative stress contributes to outcome severity in a Drosophila melanogaster model of classic galactosemia. *Dis. Model. Mech.* 6, 84-94. 10.1242/dmm.010207 22773758 PMC3529341

[DMM022988C17] Jumbo-Lucioni P., Parkinson W. and Broadie K. (2014a). Overelaborated synaptic architecture and reduced synaptomatrix glycosylation in a Drosophila classic galactosemia disease model. *Dis. Model. Mech.* 7, 1365-1378. 10.1242/dmm.017137 25326312 PMC4257005

[DMM022988C18] Jumbo-Lucioni P. P., Ryan E. L., Hopson M. L., Bishop H. M., Weitner T., Tovmasyan A., Spasojevic I., Batinic-Haberle I., Liang Y., Jones D. P. et al. (2014b). Manganese-based superoxide dismutase mimics modify both acute and long-term outcome severity in a Drosophila melanogaster model of classic galactosemia. *Antioxid. Redox Signal.* 20, 2361-2371. 10.1089/ars.2012.5122 23758052 PMC4005492

[DMM022988C19] Kushner R. F., Ryan E. L., Sefton J. M. I., Sanders R. D., Lucioni P. J., Moberg K. H. and Fridovich-Keil J. L. (2010). A Drosophila melanogaster model of classic galactosemia. *Dis. Model. Mech.* 3, 618-627. 10.1242/dmm.005041 20519569 PMC2931538

[DMM022988C20] Leslie N. D. (2003). Insights into the pathogenesis of galactosemia. *Annu. Rev. Nutr.* 23, 59-80. 10.1146/annurev.nutr.23.011702.073135 12704219

[DMM022988C21] Leslie N. D., Yager K. L., McNamara P. D. and Segal S. (1996). A mouse model of galactose-1-phosphate uridyl transferase deficiency. *Biochem. Mol. Med.* 59, 7-12. 10.1006/bmme.1996.0057 8902187

[DMM022988C22] Lubetzky R., Sever O., Mimouni F. B. and Mandel D. (2015). Human milk macronutrients content: effect of advanced maternal age. *Breastfeed. Med.* 10, 433-436. 10.1089/bfm.2015.0072 26171573

[DMM022988C23] Mummery-Widmer J. L., Yamazaki M., Stoeger T., Novatchkova M., Bhalerao S., Chen D., Dietzl G., Dickson B. J. and Knoblich J. A. (2009). Genome-wide analysis of Notch signalling in Drosophila by transgenic RNAi. *Nature* 458, 987-992. 10.1038/nature07936 19363474 PMC2988197

[DMM022988C24] Ning C., Reynolds R., Chen J., Yager C., Berry G. T., McNamara P. D., Leslie N. and Segal S. (2000). Galactose metabolism by the mouse with galactose-1-phosphate uridyltransferase deficiency. *Pediatr. Res.* 48, 211-217. 10.1203/00006450-200008000-00015 10926297

[DMM022988C25] Pyhtila B. M., Shaw K. A., Neumann S. E. and Fridovich-Keil J. L. (2015). Newborn screening for galactosemia in the United States: looking back, looking around, and looking ahead. *JIMD Rep.* 15, 79-93. 10.1007/8904_2014_302 24718839 PMC4413015

[DMM022988C26] Ross K. L., Davis C. N. and Fridovich-Keil J. L. (2004). Differential roles of the Leloir pathway enzymes and metabolites in defining galactose sensitivity in yeast. *Mol. Genet. Metab.* 83, 103-116. 10.1016/j.ymgme.2004.07.005 15464425

[DMM022988C27] Ryan E. L., DuBoff B., Feany M. B. and Fridovich-Keil J. L. (2012). Mediators of a long-term movement abnormality in a Drosophila melanogaster model of classic galactosemia. *Dis. Model. Mech.* 5, 796-803. 10.1242/dmm.009050 22736462 PMC3484862

[DMM022988C28] Ryder E. and Russell S. (2003). Transposable elements as tools for genomics and genetics in Drosophila. *Brief. Funct. Genomic. Proteomic.* 2, 57-71. 10.1093/bfgp/2.1.57 15239944

[DMM022988C29] Sanders R. D., Sefton J. M. I., Moberg K. H. and Fridovich-Keil J. L. (2010). UDP-galactose 4′ epimerase (GALE) is essential for development of Drosophila melanogaster. *Dis. Model. Mech.* 3, 628-638. 10.1242/dmm.005058 20519568 PMC2931539

[DMM022988C30] Schweitzer S., Shin Y., Jakobs C. and Brodehl J. (1993). Long-Term Outcome in 134 Patients with Galactosaemia. *Eur. J. Pediatr.* 152, 36-43. 10.1007/BF02072514 8444204

[DMM022988C31] Segal S. (1995). Galactosemia unsolved. *Eur. J. Pediatr.* 154, S97-SS102. 10.1007/BF02143813 7671976

[DMM022988C32] Spencer J. B., Badik J. R., Ryan E. L., Gleason T. J., Broadaway K. A., Epstein M. P. and Fridovich-Keil J. L. (2013). Modifiers of ovarian function in girls and women with classic galactosemia. *J. Clin. Endocrinol. Metab.* 98, E1257-E1265. 10.1210/jc.2013-1374 23690308 PMC3701263

[DMM022988C33] Tang M., Odejinmi S. I., Vankayalapati H., Wierenga K. J. and Lai K. (2012). Innovative therapy for Classic Galactosemia-tale of two HTS. *Mol. Genet. Metab.* 105, 44-55. 10.1016/j.ymgme.2011.09.028 22018723 PMC3253915

[DMM022988C34] Tang M., Siddiqi A., Witt B., Yuzyuk T., Johnson B., Fraser N., Chen W., Rascon R., Yin X., Goli H. et al. (2014). Subfertility and growth restriction in a new galactose-1 phosphate uridylyltransferase (GALT) - deficient mouse model. *Eur. J. Hum. Genet.* 22, 1172-1179. 10.1038/ejhg.2014.12 24549051 PMC4169538

[DMM022988C35] Waggoner D. D., Buist N. R. and Donnell G. N. (1990). Long-term prognosis in galactosaemia: results of a survey of 350 cases. *J. Inherit. Metab. Dis.* 13, 802-818. 10.1007/BF01800204 1706789

[DMM022988C36] Waisbren S. E., Potter N. L., Gordon C. M., Green R. C., Greenstein P., Gubbels C. S., Rubio-Gozalbo E., Schomer D., Welt C., Anastasoaie V. et al. (2012). The adult galactosemic phenotype. *J. Inherit. Metab. Dis.* 35, 279-286. 10.1007/s10545-011-9372-y 21779791 PMC3641771

[DMM022988C37] Walter J. H., Collins J. E., Leonard J. V., CHISWICK M. and MARCOVITCH H. (1999). Recommendations for the management of galactosaemia • Commentary. *Arch. Dis. Child.* 80, 93-96. 10.1136/adc.80.1.93 10325771 PMC1717786

